# Phenotypic prevalence of obesity and metabolic syndrome among an underdiagnosed and underscreened population of over 50 million children and adults

**DOI:** 10.3389/fgene.2022.961116

**Published:** 2022-09-06

**Authors:** Eric GR Kim, David C Kaelber

**Affiliations:** ^1^ The Department of Family Medicine, School of Medicine, Case Western Reserve University, Cleveland, OH, United States; ^2^ The Center for Clinical Informatics Research and Education, The MetroHealth System, Cleveland, OH, United States; ^3^ The Departments of Internal Medicine, Pediatrics, and Population and Quantitative Health Sciences, School of Medicine, Case Western Reserve University, Cleveland, OH, United States

**Keywords:** metabolic syndrome, adolescents, pediatric, obesity, epic, cosmos

## Abstract

**Background:** Metabolic syndrome is a phenotypic condition associated with a variety of genotypes. Studies of rare genotypes can be made more difficult by clinical underscreening of the population for the phenotypic traits that define metabolic syndrome to clinicians. Studies have demonstrated underdiagnosis of pediatric obesity, as well as reduced rates of pediatric screening for obesity related conditions, including conditions leading to a diagnosis of metabolic syndrome. If true, there may be a significant underdiagnosis of metabolic syndrome among the pediatric population compared to the adult population.

**Methods:** Using Epic’s Cosmos Data Network aggregated, de-identified patient data collected from healthcare organizations using the Epic electronic health record (EHR), we examined obesity and metabolic syndrome rates among adult and pediatric patients. We also examined screening rates for obesity related conditions and metabolic syndrome among adult and pediatric patients across the United States. We also sought to compare rates between subgroups within the population including age, sex, and race.

**Results:** In our population, 45% of adults and 27% of pediatric population were obese by age and gender specific BMI criteria. 38% of the obese adult population had an ICD-10 code associated with the diagnosis vs. 52% of the pediatric population. Of adults meeting obesity criteria, 36% had results for appropriate, guideline-based blood laboratory testing for insulin resistance, 40–42% for dyslipidemia, and 55% for hepatic steatosis. 36% of obese adult patients had none of the recommended blood laboratory testing. 31% of the adult population met diagnostic criteria for metabolic syndrome. Of pediatric patients meeting obesity criteria, 27% had results for appropriate blood laboratory testing for insulin resistance, 28% for dyslipidemia, and 33% for hepatic steatosis. 59% of obese pediatric patients had none of the recommended blood laboratory testing. 3% of the pediatric population met criteria for diagnosis of metabolic syndrome.

**Discussion:** This study represents one of the largest multicenter national cohorts assembled for studying metabolic syndrome (over 50 million patients) and demonstrates the power of emerging aggregated EHR tools for research. Although obesity is better diagnosed in pediatric patients than in adult patients, significantly lower screening rates for obesity related conditions occurred in pediatric patients compared to adults. Statistically significant, but clinically negligible differences in screening rates were found by race and gender. These results support smaller prior studies that suggest that obesity is under-diagnosed and obesity related conditions underscreened in pediatric and adult populations, and additionally suggests underdiagnosis of metabolic syndrome among United States pediatric and adult patients.

## 1 Introduction

Metabolic syndrome is a condition marked phenotypically by insulin resistance, dyslipidemia, hypertension, and/or obesity. Studies into the pathophysiology of metabolic syndrome have identified a cascade of effects that ultimately result in a prothrombotic and proinflammatory state that is associated with higher rates of atherosclerosis, coronary artery disease, and dermatologic conditions among other things (McCracken et al., 2018). The pathogenetic etiology of these traits has been hinted at in twin studies including the Northern Manhattan Family Study which showed significantly increased rates of obesity-related conditions, for example hypertension and diabetes, between monozygotic vs. dizygotic twins (Carmelli et al., 1994). Since that time, many genomic studies have been performed identifying a variety of candidate genes that may play a role in the phenotypic manifestations of obesity and metabolic syndrome (Stančáková and Laakso, 2014). Studies of rare metabolic syndrome related genotypes can be made easier when large samples of the phenotype are available through large data networks. However, phenotypic population discovery can still be difficult or biased if measures used to define the phenotype are unevenly screened in the clinical environment.

The International Diabetes Federation (IDF) Task Force on Epidemiology defines adult metabolic syndrome as a combination of any 3 of 5 criteria: 1) triglycerides above 150 mg/dl, 2) fasting glucose greater than 100 mg/dl, 3) reduced high density lipoprotein (HDL) below gender specific thresholds, 4) systolic blood pressure above 130 mmHg or diastolic above 85 mmHg, and 5) abnormal waist circumference when compared to gender specific thresholds. Treatment for any of these conditions may also be used as criteria (Zimmet et al., 2007). A 2017 analysis of data from the National Health and Nutrition Examination Survey demonstrated the prevalence of metabolic syndrome in the United States was 34.2%. Metabolic syndrome has had an increasing prevalence in the adult population in the United States over the past few decades (Moore et al., 2017).

The definition of pediatric metabolic syndrome is less clear, and multiple definitions have been used (Weihe and Weihrauch-Blüher, 2019; Serbis et al., 2020). The International Diabetes Federation defines pediatric metabolic syndrome between ages 10 and 16 by similar criteria to the adult syndrome, with the exception of replacing waist circumference with waist circumference percentile for age and sex compared to the national population (Zimmet et al., 2007). Several studies in relatively small populations have demonstrated underdiagnosis of pediatric obesity (Benson et al., 2009), as well as reduced rates of pediatric screening for obesity related conditions (Benson et al., 2011), including conditions that would satisfy criteria for the diagnosis of metabolic syndrome. It is not clear from the literature if there is an underdiagnosis of metabolic syndrome due to underscreening for obesity related conditions. Given that the prevalence of obesity among pediatric patients is around 20% (Benson et al., 2009; Cardel et al., 2020), metabolic syndrome could affect a significant portion of the pediatric population.

## 2 Methods

### Epic cosmos data network

The Epic Systems electronic health record (EHR) has significant market representation in the United States. As of the 2021 KLAS Research report, the Epic EHR is present in 31% of United States hospitals and 42% of United States hospital beds (KLAS, 2021). The Epic Cosmos Data Network was developed as an aggregation of de-identified patient information from healthcare systems using Epic EHRs across the United States who voluntarily submit their data (Tarabichi et al., 2021). Cosmos currently contains the records of over 140 million unique patients across 50 states, 800 + hospitals, and 10,000 clinics (Softare & Services, 2022). This de-identified data is available for summative query, including by total patient count meeting particular criteria.

From the Epic Cosmos population, we examined rates of obesity and metabolic syndrome, as well as screening rates for obesity related conditions and metabolic syndrome among adult and pediatric patients across the United States. We also compared rates between subgroups within the dataset population including age, sex, and race.

### Study design

We performed a cross-sectional analysis to identify the characteristics of all patients with obesity, based on body mass index (BMI) criteria. In this study, those with a current age from 10 to 17 years old were used to represent the pediatric population due to the absence of a clear definition for pediatric metabolic syndrome below 10 years old (Zimmet et al., 2007). Patients with current age 18 years old and older were considered adults. Since BMI thresholds for obesity vary with age and gender, measured BMI values were restricted to appropriate age ranges within subqueries. Among patients ≥10 years old, all patients with a BMI recorded within the appropriate age range between 11/25/2018 and 11/24/2021 were sampled (n = 55,042,652). A 3-years lookback was selected as a reasonable balance between clinical relevance and completeness of the data. Of these patients, those with a BMI recorded within the appropriate age rage above the threshold for the diagnosis of obesity were counted. For adults, an obesity BMI threshold of ≥30 kg/m^2^ was used. For pediatric patients, a gender and age specific BMI threshold was used in place of BMI percentile, as BMI percentile was not a searchable data value. CDC growth chart data for the 95th percentile BMI for males and separately for females was averaged for each year between 10 and 17 years of age. For example, for a 15-year-old boy BMIs in the 95th percentile ranged from 26.45 kg/m^2^ to 27.15 kg/m^2^. The average of the upper and lower bounds of this range, 26.8 kg/m^2^, was rounded to 27 kg/m^2^ and used as the BMI cut-off. These annual averages were used as the gender and age specific thresholds for pediatric obesity in this analysis. Each pediatric age-gender group was queried individually, and results were aggregated.

Those patients who met criteria for obesity above were then queried for an ICD-10 obesity diagnosis included under the SNOMED CT concept “obesity”. The proportion of patients with an obesity diagnosis who had a BMI that crossed the threshold for obesity was determined for both the adult and pediatric populations.

**FIGURE 1:**
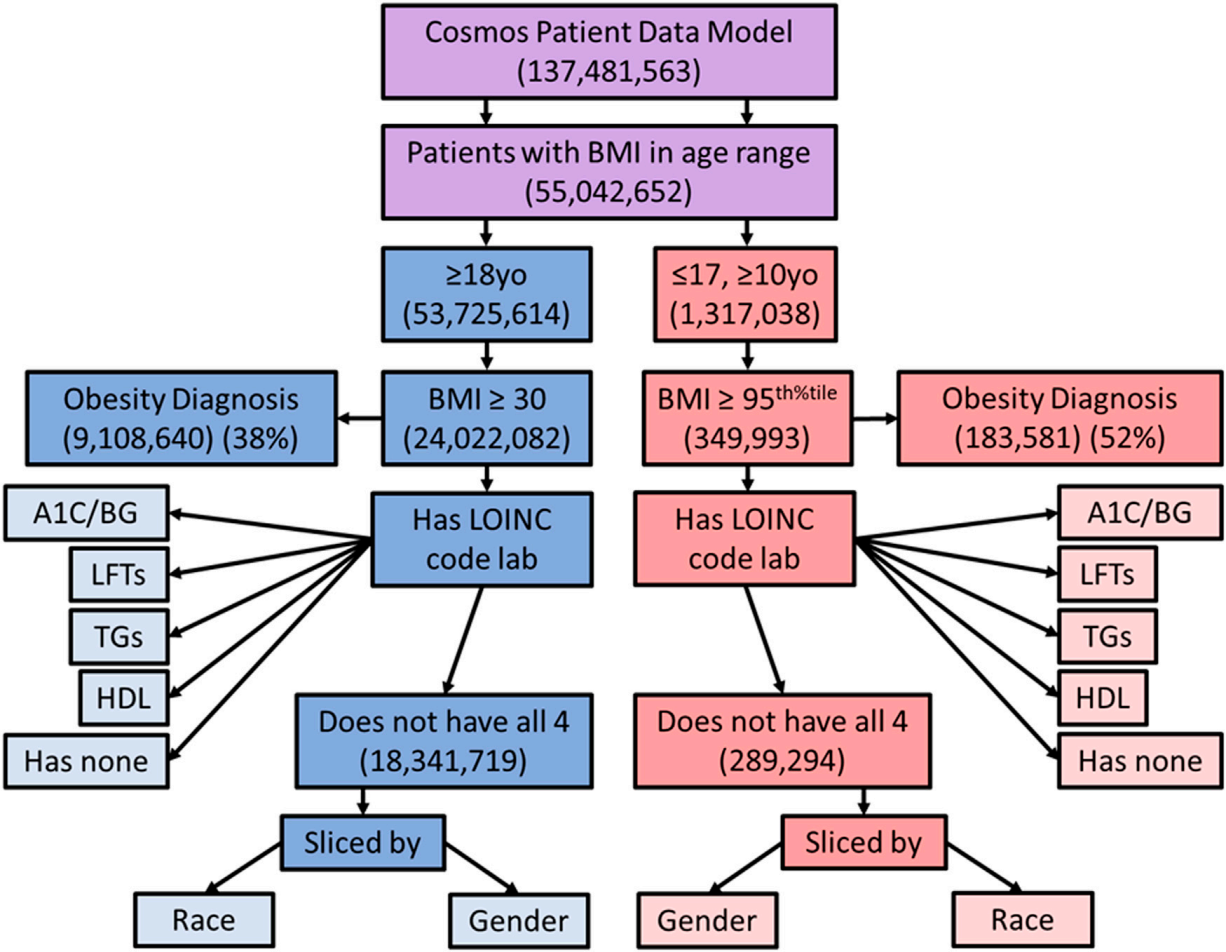
Diagram showing the way in which the sample population was sliced for comparison. Purple represents the combined population, blue adults, and red pediatric patients. yo = years old, BMI = body mass index, LOINC = Logical Observation Identifiers Names and Codes, A1C = hemoglobin A1C, LFTs = liver function tests, TGs = triglycerides, BG = blood glucose, HDL = high density lipoproteins.

In order to explore the appropriate blood laboratory testing for metabolic syndrome, we searched the population of patients who met BMI criteria for obesity to see how many also had blood laboratory values for high density lipoprotein (HDL) and triglycerides (TGs) screening for dyslipidemia, fasting blood glucose (FBG) or hemoglobin A1C screening for insulin resistance, and/or hepatic transaminases (LFTs) for screening for hepatic steatosis, within the lookback range, based on established metabolic syndrome evaluation guidelines (Garvey et al., 2016). The total proportion of obese patients, by BMI criteria, that had been screened by each lab individually was determined. In addition, the proportion of obese patients without all 4 blood laboratory results on record and without any of the blood laboratory results on record were determined. These proportions were compared between male and female patients, adult and pediatric patients, and patients by race, to identify disparities. Figure 1 depicts the manner in which the original population was sub-divided by queries.

To explore the prevalence of metabolic syndrome we also queried the dataset for patients meeting metabolic syndrome criteria. All patient records with a BMI recorded within the appropriate age range between 6/23/2021 and 12/15/2021 were queried (n = 25,381,916). We limited this to a clinically meaningful 6-months look-back period. Waist circumference, one of the criteria for metabolic syndrome, is not routinely measured and was not available. Previous studies have found a strong correlation between BMI and waist circumference (Ryan et al., 2008; Gierach et al., 2014). In this study, we used elevated BMI >30 kg/m^2^ in patients ≥16 years old or BMI corresponding to the ≥90th percentile in patients between 10 and 15 years old as a proxy for the waist circumference criteria in diagnosis of metabolic syndrome. Systolic blood pressure above 130, diastolic blood pressure above 85, abnormal HDL, abnormal TGs, and random blood glucose above 200, and abnormal fasting glucose/hemoglobin A1c blood laboratory results were also used as criteria. Record of prescribed medications for dyslipidemia, impaired glucose tolerance, and hypertension were also used as equivalent criteria for each of the above, consistent with the definition of metabolic syndrome. All patients with a BMI recorded within the appropriate age range were searched for meeting at least 3 of the 5 above criteria. The proportion of the adult and pediatric populations with a BMI on record that also met at least 3 of 5 criteria for a metabolic syndrome diagnosis was determined and compared.

Statistics were performed on resulting patient counts between each sub-group. Given the significant population and sub-group size, most statistical tests were expected to return a significant result. χ^2^ tests for heterogeneity were determined to be the most appropriate tests for this data. The χ^2^ test for heterogeneity was applied to several different comparisons: 1) the population of adult and pediatric patients with a BMI classification as obese compared to the population of the same without, 2) the population of adult and pediatric patients who were obese by BMI criteria with an obesity diagnosis compared to the population of the same without, 3) the population of adult and pediatric patients who were obese by BMI criteria with each individual screening lab, no screening labs, and some but not all screening labs compared to the population of the same without, 4) the population of male and female patients who were obese by BMI criteria with each individual screening lab compared to the population of the same without, 5) the population of patients of various races who were obese by BMI criteria with each individual screening lab compared to the population of the same without, and 6) the population of adult and pediatric patients meeting criteria for metabolic compared to the same without.

### Queries

HDL, TGs, BGs, and LFTs were queried for “final” and “abnormal” status and used as criteria. Available random blood glucose results above 200 were classified as abnormal and those cases also counted. Table 1 summarizes the lab components that were used. For medications used for treatment, RxNorm medication groupers based on medication class (for example “antihyperglycemics”) were used to search for medications in patient records. Table 2 summarizes the medication groups that were used.

**TABLE 1:** LOINC code laboratory components used in each criterion.

Criterion	LOINC code lab component
High Density Lipoprotein	CHOLESTEROL IN HDL (MASS/VOLUME) IN SERUM OR PLASMA 2085-9
	CHOLESTEROL IN HDL (MOLES/VOLUME) IN SERUM OR PLASMA 14646-4
	CHOLESTEROL IN HDL (MASS OR MOLES/VOLUME) IN SERUM OR PLASMA 35197-3
Triglycerides	TRIGLYCERIDE (MASS/VOLUME) IN BLOOD 3043-7
	TRIGLYCERIDE (MASS/VOLUME) IN SERUM OR PLASMA 2571-8
	TRIGLYCERIDE (MOLES/VOLUME) IN SERUM OR PLASMA 14927-8
	TRIGLYCERIDE (MASS/VOLUME) IN SERUM OR PLASMA -FASTING 3048-6
	TRIGLYCERIDE (MASS/VOLUME) IN SERUM OR PLASMA -12 HOURS FASTING 1644-4
Fasting Blood Glucose/Hemoglobin A1C	FASTING GLUCOSE (MASS/VOLUME) IN VENOUS BLOOD 1557-8
	FASTING GLUCOSE (MASS/VOLUME) IN CAPILLARY BLOOD 1556-0
	FASTING GLUCOSE (MASS/VOLUME) IN SERUM OR PLASMA 1558-6
	FASTING GLUCOSE (MOLES/VOLUME) IN SERUM OR PLASMA 14771-0
	FASTING GLUCOSE (MASS OR MOLES/VOLUME) IN SERUM OR PLASMA 35184-1
	FASTING GLUCOSE (MASS/VOLUME) IN CAPILLARY BLOOD BY GLUCOMETER 41604-0
	FASTING GLUCOSE (MOLES/VOLUME) IN CAPILLARY BLOOD BY GLUCOMETER 14770-2
	GLUCOSE (MASS/VOLUME) IN SERUM OR PLASMA -8 HOURS FASTING 17865-7
	PHENX - FASTING PLASMA GLUCOSE FOR DIABETES SCREENING - BLOOD DRAW PROTOCOL 140801 62851-1
	A1C (Grouper: Cosmos Core)
	HEMOGLOBIN A1C (MASS/VOLUME) IN BLOOD 41995-2
Hepatic Transaminases	ALANINE AMINOTRANSFERASE (ENZYMATIC ACTIVITY/VOLUME) IN BLOOD 76625-3
	ALANINE AMINOTRANSFERASE (ENZYMATIC ACTIVITY/VOLUME) IN SERUM OR PLASMA 1742-6
	ALANINE AMINOTRANSFERASE (ENZYMATIC ACTIVITY/VOLUME) IN SERUM, PLASMA OR BLOOD 77144-4
	ASPARTATE AMINOTRANSFERASE (PRESENCE) IN SERUM OR PLASMA 27344-1
	ASPARTATE AMINOTRANSFERASE (ENZYMATIC ACTIVITY/VOLUME) IN SERUM OR PLASMA 1920-8

**TABLE 2:** Medication RxNorm groups used in each criterion.

Treatment for	Medication grouper
Hypertension	ANTIHYPERTENSIVES, ACE INHIBITOR/DIETARY SUPP.COMB
	ANTIHYPERTENSIVES, ANGIOTENSIN RECEPTOR ANTAGONIST
	ANTIHYPERTENSIVES, GANGLIONIC BLOCKERS
	ANTIHYPERTENSIVES, ACE INHIBITORS
	ANTIHYPERTENSIVES, SYMPATHOLYTIC
	ANTIHYPERTENSIVES, MISCELLANEOUS
	ANTIHYPERTENSIVES, VASODILATORS
	Diuretic - Thiazides and Related
Insulin Resistance	ANTIHYPERGLYCEMICS
Dyslipidemia (HDL)	Antihyperlipidemic - Fibric Acid Derivatives
	Antihyperlipidemic - HMG CoA Reductase Inhibitors (statins)
	Antihyperlipidemic - Omega-3 Fatty Acid Type
Dyslipidemia (TGs)	Antihyperlipidemic - Fibric Acid Derivatives
	Antihyperlipidemic - Omega-3 Fatty Acid Type
	Antihyperlipidemic - HMG CoA Reductase Inhibitor and Niacin Comb

Because this study used tools that only allowed access to aggregated, population-level, de-identified data, no individual protected health information was accessed. Therefore, Institutional Board Review was not sought or obtained.

## 3 Results

Baseline analysis of the Epic Cosmos patient population is shown in Table 3. The population of patients studies was 53.4% male and 46.5% female. Patient identified race was 62.9% white, 14.1% black, 3.7% Asian, 0.6% American Indian or Alaska Native, and 0.5% Native Hawaiian or Pacific Islander. 7.6% of patients identified their race as other. 13.3% of patients did not have race data. There were more adults than children at 18.8% pediatric age and 81.2% adult age patients.

**TABLE 3:** Baseline characteristics of study population.

	Number of Patients
Total	137,481,563
Gender	
Male	73,380,581
Female	63,965,593
Race	
White	86,469,293
Black	19,316,851
Asian	5,071,677
American Indian or Alaska Native	873,283
Native Hawaiian or Other Pacific Islander	701,545
Other Race	10,385,385
None of the Above	18,299,644
Age (years)	
0−<10	13,865,677
10−<18	11,940,917
18−<30	21,335,708
30−<50	34,802,257
50−<70	34,009,637
≥70	21,526,715
BMI	
0−<20 kg/m^2^	12,908,671
20−<30 kg/m^2^	36,852,285
30−<40 kg/m^2^	21,300,955
≥40 kg/m^2^	6,003,390

For our 3-years analysis of the adult group, 45% of the population with a BMI value met criteria for obesity with BMI ≥30 kg/m^2^ 38% of the obese population had an ICD-10 code associated with the diagnosis. Of those that met criteria for obesity, 36% had blood laboratory results appropriate to screen for insulin resistance, 40–42% for dyslipidemia, and 55% for hepatic steatosis. 36% of obese adult patients had none of the recommended blood laboratory testing for metabolic syndrome. Based on our analysis, 31% of the adult population with a BMI value on record met criteria for the diagnosis of metabolic syndrome, although 76% of the obese adult population was not screened or only incompletely screened.

Likewise, for the pediatric group, 27% of the population with a BMI value met criteria for obesity with BMI corresponding to the ≥95th percentile. 52% of the obese population had an obesity associated ICD-10 diagnosis code. Of those that met criteria for obesity, 27% had blood laboratory results appropriate to screen for insulin resistance, 28% for dyslipidemia, and 33% for hepatic steatosis. 59% of obese pediatric patients in the analysis had none of the recommended blood laboratory testing for metabolic syndrome. Based on our analysis, 3% of the pediatric population with a BMI value met criteria for diagnosis of metabolic syndrome, although 83% of obese pediatric population were not screened or only incompletely screened.

Differences in blood laboratory testing for metabolic syndrome among obese patients were evaluated for gender and race. Among adult patients, 75% of the obese male population vs. 77% of the obese female population were underscreened for metabolic syndrome based not having all appropriate blood laboratory tests. Among pediatric patients, 84% of the obese male population vs. 82% of the obese female population were underscreened. For obese adult patients, 76% of white patients, 76% of black patients, 72% of Asian patients, 76% of American Indian or native Alaskan patients, and 73% of native Hawaiian or pacific islander patients were underscreened. For obese pediatric patients, 84% of white patients, 81% of black patients, 82% of Asian patients, 79% of American Indian or native Alaskan patients, and 80% of native Hawaiian or pacific islander patients were underscreened. This data is presented in more detail in Table 4.

**TABLE 4:** Summary of patient populations. Using χ^2^ test for heterogeneity, *p* = <0.001 for comparisons described in the methods section.

Characteristic	Adult	Pediatric	χ^2^ *p* Value
BMI consistent with obesity	45% (24,022,082/53,725,614)	27% (349,993/1,317,038)	<0.001
Obesity BMI with diagnosis of obesity	38% (9,108,640/24,022,082)	52% (183,581/349,993)	<0.001
Among patients with BMI in obese range			
HDL performed	40% (9,676,508/24,022,082)	28% (97,943/349,993)	<0.001
TG performed	42% (10,000,474/24,022,082)	28% (98,367/349,993)	<0.001
Fasting BG or A1C performed	36% (8,614,323/24,022,082)	27% (94,790/349,993)	<0.001
LFTs performed	55% (13,245,496/24,022,082)	33% (115,045/349,993)	<0.001
Without all 4 metabolic syndrome labs performed	76% (18,341,719/24,022,082)	83% (289,294/349,993)	<0.001
With no metabolic syndrome labs performed	36% (8,674,884/24,022,082)	59% (204,788/349,993)	<0.001
Among obese patients without all 4 metabolic syndrome labs performed			
Males	75% (7,521,573/10,025,710)	84% (156,810/187,670)	<0.001
Females	77% (10,820,146/13,996,372)	82% (132,484/162,323)	<0.001
American Indian or Native Alaskan	76% (141,064/185,647)	79% (2,967/3,773)	<0.001
Asian	72% (269,805/374,513)	82% (5,937/7,272)	<0.001
Black	76% (3,291,466/4,329,025)	81% (59,550/73,102)	<0.001
Native Hawaiian or Pacific Islander	73% (79,078/108,787)	80% (1,931/2,425)	<0.001
Other	77% (1,277,089/1,656,359)	77% (29,094/37,546)	<0.001
White	76% (13,041,709/17,167,747)	84% (180,556/214,938)	<0.001
Race Not Recorded	82% (850,580/1,040,029)	81% (24,400/30,261)	<0.001
Meeting 3+ of 5 criteria for metabolic syndrome diagnosis	31% (7,603,048/24,317,964)	3% (34,364/1,063,952)	<0.001

## 4 Discussion

This study evaluates metabolic syndrome among over 50 million patients and demonstrates the power of emerging aggregated EHR tools for research. The rising burden of obesity and metabolic syndrome has previously been noted (Moore et al., 2017). This study seeks to provide a cross-sectional look into a very large proportion of the United States population to determine obesity, metabolic syndrome, and metabolic syndrome blood laboratory testing rates, as well as whether there are differences in these rates based on age, sex, and race.

BMI is typically the first sign noted by medical providers that can trigger further investigation including blood laboratory testing for criteria of metabolic syndrome. It can be easily determined at any office visit with a simple measurement of height and weight. Blood pressure is similarly easy to measure at any office visit. However, to obtain other criteria for metabolic syndrome in patients, typically more invasive blood laboratory tests must be run for HDL, TGs, and insulin resistance (by blood glucose or HgA1C). As a result, in primary care, it is reasonable to engage in a two-step screening process. BMI acts as the first screening. For those who meet criteria for obesity, a second set of screening tests may be performed to screen for comorbid conditions, as recommended by the American College of Endocrinology (Garvey et al., 2016). This may include conditions such as dyslipidemia, insulin resistance, or hepatic steatosis. In this study, we extracted a subpopulation from the larger Epic Cosmos Data Network of patients who met criteria for obesity. We examined them for evidence of further blood laboratory testing for comorbid conditions and metabolic syndrome. We also examined age, sex, and race for possible disparities in screening and evaluation.

Among patients with a BMI on record, we showed 45% of adults and 27% of pediatric patients had a BMIs consistent with obesity. This is slightly above previously published values for obesity which average between 30 and 40% for adults (Inoue et al., 2018; Stierman et al., 2020), and around 20% for pediatric patients (Benson et al., 2009; Cardel et al., 2020; Stierman et al., 2020).

Among obese patients, rates of blood laboratory testing for obesity related complications (including criteria for metabolic syndrome) were lower for pediatric patients than for adult patients. This was true for all individual blood laboratory test screening rates (HDL, TGs, blood glucose or A1C, LFTs), as well as for the absence of screening which showed 36% of adults had none of the recommended metabolic syndrome screening related blood laboratory tests compared to 59% of pediatric patients. These numbers suggest significant underscreening among obese pediatric patients compared to adults. This conclusion supports previous data that suggests underscreening of pediatric patients for obesity related conditions (Benson et al., 2011). Blood laboratory testing for metabolic syndrome in obese individuals did vary by race and gender in a statistically significant manner, suggesting racial and gender disparities in clinical care of obesity and metabolic syndrome. However, these differences in screening rates (1–10%) were small compared to the difference in screening rate between adult and pediatric patients, and in some cases may be clinically negligible. Additionally, the differences in screening rates between race and gender sub-populations were significantly smaller than the overall underscreening rate for each sub-population individually.

Using BMI/BMI percentile as a proxy for waist circumference to define metabolic syndrome, 31% of adult patients and 3% of pediatric patients with a BMI on record had metabolic syndrome. This metabolic syndrome rate among adults is concordant with previous studies performed on the prevalence of metabolic syndrome which showed a 34% prevalence (Moore et al., 2017; Hirode and Wong, 2020). Likewise, the 3% prevalence of metabolic syndrome among pediatric patients is concordant with research which suggests a 3.3% prevalence (Friend et al., 2013; Al-Hamad and Raman, 2017), though the estimates of pediatric metabolic syndrome prevalence vary widely due to inconsistent definitions. However, given the evidence above that obese pediatric patients are significantly underscreened for metabolic syndrome, there is a risk that metabolic syndrome is significantly underdiagnosed in this population.

The χ^2^ test for heterogeneity returned *p*-values <0.001 for all comparisons described in the methods section. As anticipated, the large sample sizes in this study tend to drive statistical testing towards significance. While technically statistically significant, the difference in screening rates between genders and races are very small, and may not be clinically significant. By comparison, screening rates between adult and pediatric populations show large differences that are both statistically and clinically significantly meaningful.

Despite the steady rise in pediatric obesity within the United States over the past several decades, it remains lower than the prevelance of obesity in the adult population (Benson et al., 2009; Cardel et al., 2020). Obesity related conditions like the group of diagnoses that make up metabolic syndrome are more common in obese adults (Friend et al., 2013; Al-Hamad and Raman, 2017; Moore et al., 2017). Given these differences, clinicians may reasonably shift more attention to the treatment of obesity and metabolic syndrome in adults. This may be a reason for reduced rates of screening for obesity related conditions among children. Additionally, the definition of obesity is less easily measured in a growing pediatric patient than it is in an adult patient. Instead of a simple BMI calculation, pediatric patients must have their BMI assessed in comparison to standard CDC population growth charts (Cheung et al., 2016). The additional complexity that this requires may present a barrier that reduces the rate of recognition by clinicians. Finally, both clinican and parental acceptance of recommended blood tests for pediatric patients faces strong resistance from unwilling and afraid children who are terrified of needles. Combined with pressure from national campaigns to appropriately reduce low-value blood laboratory testing (Hiscock et al., 2018), it is possible that blood tests are simply less likely to be ordered and completed in children than they are in adults.

Systemic challenges may also contribute to lower screening rates among children. Phlebotomists that have additional skill and experience in venipuncture with pediatric patients are likely less common than those who do not, further increasing the barrier to completing an ordered pediatric blood test. Additionally, even if a clinician orders a blood test with intent to screen a pediatric patient, protocol varies between clinics and may present another barrier to actual completion of the order. Some clinics perform phlebotomy to complete ordered blood tests while patients are in the clinic, while others require the patient to travel to a separate collection laboratory or phlebotomy site. It is likely that some patients will not perform this second step, both among adult and pediatric patients. However, it may be more common among pediatric patients due to other factors such as parental schedules, school schedules, and the resistance to getting the test done as mentioned above. The population that was analyzed in this study only sampled completed blood tests, and so may have failed to quantify incomplete blood test orders that would have demonstrated an intent to screen.

Confounding non-medical factors may have influenced the detected difference in screening rates between adults and children in our dataset population. The aggregated dataset relies on contributions from individual organizations that use the Epic EHR. Contributions are voluntary, and only include the years during which an organization was using the Epic EHR. The full spectrum of pediatric care may not be as well represented in this record compared to adult care. However, the baseline characteristics of the dataset population suggest that the proportion of pediatric patients is only a little less than the demographics of the United States population, suggesting that if a confounding factor is present, it is minimal. Any further confounding effect from this phenomenon is likely normalized by our use of proportions and not absolute patient counts.

Several limitations of this study should be considered. The population that was used shows similar characteristics to the United States population. However, there was a significant proportion of the population that did not have a race identifier on record (13%). This was also true of other relevant data like BMI (48% of adult patients did not have a recorded BMI), which lead to these patients being excluded from the analysis. Despite these exclusions, there was still tens of millions of patients available for analysis, presumably minimizing the effect of any bias from this source. In addition, the database that was used represents a biased selection of EHRs across the nation, likely over-representing the patients that would be seen at larger medical systems where EHRs are more prevalent. These potential sources of bias may have made our population less representative of the United States population as a whole. Despite this risk, the population that was analyzed in this study showed characteristics remarkably similar to that of other published results, including similar rates of adult metabolic syndrome and obesity. This study was additionally limited by the 3-years look back for obesity screening data, and 6 months look back for metabolic syndrome data. This truncation leaves open the possibility that BMI, medication orders, diagnoses, and laboratory tests occurring earlier than this period may not have been captured in the analysis leading to underestimates. However, from a clinical perspective, obesity and metabolic screening of obese patients should typically occur in these time windows for good clinical care. Finally, this study was also limited by the need to use laboratory results flagged as “abnormal” instead of the specific thresholds enumerated in diagnostic guidelines for metabolic syndrome. However, the reference range that determines “abnormal” for most labs is likely similar to diagnostic thresholds.

## 5 Conclusion

In this study, we used aggregated, population level EHR data among over 50 million patients to study a large proportion of the United States population to provide cross-sectional data on the prevalence of obesity and metabolic syndrome, as well as examine the rate at which metabolic syndrome is screened for among obese populations. Obesity in the primary care environment is readily identified during routine office visits using BMI. In order to find and manage obesity related conditions and metabolic syndrome in patients, secondary screening for dyslipidemia, insulin resistance, and hypertension should be performed after identifying obesity in an individual. This study identifies a significant difference in secondary screening rates between pediatric patients and adults that meet criteria for obesity. Underdiagnosis of metabolic syndrome becomes a significant risk when screening rates are low. Despite the significantly lower prevalence of metabolic syndrome in pediatric patients compared to adults both in this population and published in literature, it is possible that the prevalence may in fact be artificially diminished due to underscreening. Given the prevalence of obesity in both adult and pediatric populations, clinical treatment of obesity related conditions such as metabolic syndrome is an imperative for the United States healthcare system. This study suggests that an important step towards this goal will be increasing rates of screening for obesity related conditions for all obese patients (based on BMI), with a particular focus on improving screening rates among pediatric patients. Further studies will be needed to confirm underscreening, elucidate disparities, and provide additional targets for focused screening efforts. Further characterizing the phenotypic population of metabolic syndrome through improved clinical screening will be an important step in improving the availability of sample populations for genomic study of metabolic syndrome.

## Data Availability

The datasets analyzed for this study can be found in Epic’s Cosmos Data Network. Please reference https://cosmos.epic.com for more information.
